# Supplementary material for: The prognostic value of Th17/Treg cell in cervical cancer: a systematic review and meta-analysis
Source: Front Oncol. 2024 Sep 11;14:1442103. doi: 10.3389/fonc.2024.1442103 (PMC11422014; doi:10.3389/fonc.2024.1442103)
Supplement: Supplementary file 1 [file DataSheet1.docx]

Supplementary Material

# Supplementary Doc 1

**The search terms in PUBMED**

((((((((((((((((((Th17 Cells[MeSH Terms]) OR (Th17 Cell[Title/Abstract])) OR (Cell, Th17[Title/Abstract])) OR (Cells, Th17[Title/Abstract])) OR (T Helper 17 Cell[Title/Abstract])) OR (TH-17 Cell[Title/Abstract])) OR (TH 17 Cell[Title/Abstract])) OR (T Helper 17 Cells[Title/Abstract])) OR (Type 17 Helper T Cells[Title/Abstract])) OR (TH-17 Cells[Title/Abstract])) OR (Cell, TH-17[Title/Abstract])) OR (Cells, TH-17[Title/Abstract])) OR (TH 17 Cells[Title/Abstract])) OR (Type 17 Helper T Cell[Title/Abstract])) OR (helper cell type 17[Title/Abstract])) OR (helper cell type 17[Title/Abstract])) OR (T helper type 17[Title/Abstract])) OR ((((((((((((((((((((((((((((((((((((((((((((((((T-Lymphocytes, Regulatory[MeSH Terms]) OR (T Lymphocytes, Regulatory[Title/Abstract])) OR (Regulatory T-Lymphocyte[Title/Abstract])) OR (Regulatory T Lymphocyte[Title/Abstract])) OR (T-Lymphocyte, Regulatory[Title/Abstract])) OR (Treg Cell[Title/Abstract])) OR (Cell, Treg[Title/Abstract])) OR (Cells, Treg[Title/Abstract])) OR (Regulatory T-Lymphocytes[Title/Abstract])) OR (Regulatory T Lymphocytes[Title/Abstract])) OR (T-Cells, Regulatory[Title/Abstract])) OR (Treg Cells[Title/Abstract])) OR (Regulatory T-Cells[Title/Abstract])) OR (Regulatory T Cell[Title/Abstract])) OR (Cell, Regulatory T[Title/Abstract])) OR (Cells, Regulatory T[Title/Abstract])) OR (Regulatory T Cells[Title/Abstract])) OR (T Cell, Regulatory[Title/Abstract])) OR (T Cells, Regulatory[Title/Abstract])) OR (Regulatory T-Cell[Title/Abstract])) OR (Th3 Cells[Title/Abstract])) OR (Cell, Th3[Title/Abstract])) OR (Cells, Th3[Title/Abstract])) OR (Th3 Cell[Title/Abstract])) OR (Suppressor T-Lymphocytes, Naturally-Occurring[Title/Abstract])) OR (Naturally-Occurring Suppressor T-Lymphocyte[Title/Abstract])) OR (Naturally-Occurring Suppressor T-Lymphocytes[Title/Abstract])) OR (Suppressor T Lymphocytes, Naturally Occurring[Title/Abstract])) OR (Suppressor T-Lymphocyte, Naturally-Occurring[Title/Abstract])) OR (Suppressor T-Cells, Naturally-Occurring[Title/Abstract])) OR (Naturally-Occurring Suppressor T-Cell[Title/Abstract])) OR (Naturally-Occurring Suppressor T-Cells[Title/Abstract])) OR (Suppressor T Cells, Naturally Occurring[Title/Abstract])) OR (Suppressor T-Cell, Naturally-Occurring[Title/Abstract])) OR (T-Cell, Naturally-Occurring Suppressor[Title/Abstract])) OR (T-Cells, Naturally-Occurring Suppressor[Title/Abstract])) OR (Tr1 Cells[Title/Abstract])) OR (Tr1 Cell[Title/Abstract])) OR (Cell, Tr1[Title/Abstract])) OR (Cells, Tr1[Title/Abstract]) ) OR (immunoregulatory T cell[Title/Abstract])) OR (immunoregulatory T cells[Title/Abstract])) OR (immunoregulatory T lymphocyte[Title/Abstract])) OR (T regulatory cell[Title/Abstract])) OR (T regulatory cells[Title/Abstract])) OR (T regulatory lymphocyte[Title/Abstract])) OR (Treg[Title/Abstract])) OR (Tregs[Title/Abstract]))) AND (((((((((((((((((((((((((((((((((((((((((((((((((((((((((((((((((((((((((((((Uterine Cervical Neoplasms[MeSH Terms]) OR (Cervical Neoplasm, Uterine[Title/Abstract])) OR (Neoplasm, Uterine Cervical[Title/Abstract])) OR (Uterine Cervical Neoplasm[Title/Abstract])) OR (Neoplasms, Cervical[Title/Abstract])) OR (Cervical Neoplasms[Title/Abstract])) OR (Cervical Neoplasm[Title/Abstract])) OR (Neoplasms, Cervix[Title/Abstract])) OR (Cervix Neoplasm[Title/Abstract])) OR (Cancer of the Uterine Cervix[Title/Abstract])) OR (Cancer of the Cervix[Title/Abstract])) OR (Cervical Cancer[Title/Abstract])) OR (Cancer, Cervical[Title/Abstract])) OR (Cervical Cancers[Title/Abstract])) OR (Uterine Cervical Cancer[Title/Abstract])) OR (Cancer, Uterine Cervical[Title/Abstract])) OR (Cervical Cancer, Uterine[Title/Abstract])) OR (Uterine Cervical Cancers[Title/Abstract])) OR (Cancer of Cervix[Title/Abstract])) OR (Cervix Cancer[Title/Abstract])) OR (Cancer, Cervix[Title/Abstract]) ) OR (cervical neoplasia[Title/Abstract])) ) OR (cervical tumor[Title/Abstract])) OR (cervical tumorigenesis[Title/Abstract])) OR (cervical tumour[Title/Abstract])) OR (cervix neoplasia[Title/Abstract])) OR (cervix tumor[Title/Abstract])) OR (cervix tumorigenesis[Title/Abstract])) OR (cervix tumour[Title/Abstract])) OR (cervix uteri tumor[Title/Abstract])) OR (neoplasia of the cervix[Title/Abstract])) OR (neoplasm of the cervix[Title/Abstract])) OR (neoplastic cervical[Title/Abstract])) OR (neoplastic cervix[Title/Abstract])) OR (tumor of the cervix[Title/Abstract])) OR (tumor of the uterine cervix[Title/Abstract])) OR (tumour of the cervix[Title/Abstract])) OR (tumour of the uterine cervix[Title/Abstract])) OR (uterine cervical neoplasia[Title/Abstract])) OR (uterine cervical neoplasms[Title/Abstract])) OR (uterine cervical tumor[Title/Abstract])) OR (uterine cervix neoplasia[Title/Abstract])) OR (uterine cervix neoplasm[Title/Abstract])) OR (uterine cervix tumour[Title/Abstract])) OR (uterine cervix tumor[Title/Abstract])) OR (CIN[Title/Abstract])) OR (Uterine Cervical Dysplasia[MeSH Terms])) ) OR (Cervical Dysplasia, Uterine[Title/Abstract])) OR (Dysplasia, Uterine Cervical[Title/Abstract])) OR (Dysplasia of Cervix Uteri[Title/Abstract])) OR (Cervix Uteri Dysplasia[Title/Abstract])) OR (Cervix Uteri Dysplasias[Title/Abstract])) OR (Cervical Intraepithelial Neoplasia[Title/Abstract])) OR (Cervical Intraepithelial Neoplasms[Title/Abstract])) OR (Cervical Intraepithelial Neoplasm[Title/Abstract])) OR (Intraepithelial Neoplasm, Cervical[Title/Abstract])) OR (Intraepithelial Neoplasms, Cervical[Title/Abstract])) OR (Neoplasm, Cervical Intraepithelial[Title/Abstract])) OR (Neoplasms, Cervical Intraepithelial[Title/Abstract])) OR (Intraepithelial Neoplasia, Cervical[Title/Abstract])) OR (Neoplasia, Cervical Intraepithelial[Title/Abstract])) OR (Cervical Dysplasia[Title/Abstract])) OR (Cervical Dysplasias[Title/Abstract])) OR (Dysplasia, Cervical[Title/Abstract])) OR (Cervix Dysplasia[Title/Abstract])) OR (Dysplasia, Cervix[Title/Abstract])) OR (Cervical Intraepithelial Neoplasia, Grade III[Title/Abstract])) OR (cervix dysplasia, uterine[Title/Abstract])) OR (dysplasia of the uterine cervix[Title/Abstract])) OR (uterine cervix squamous dysplasia[Title/Abstract])) OR (uterine cervix dysplasia[Title/Abstract])) OR (Squamous Intraepithelial Lesions of the Cervix[Title/Abstract])) OR (squamous intraepithelial cervical lesion[Title/Abstract])) OR (squamous intraepithelial lesions of the cervix[Title/Abstract])) OR (squamous intraepithelial lesion of the cervix[Title/Abstract]))

**The search terms in EMBASE**

| #3 | #1 AND #2 |
| --- | --- |
| #2 | 'immunoregulatory t cell'/exp OR 'immunoregulatory t cell' OR 'immunoregulatory t cells'/exp OR 'immunoregulatory t cells' OR 'immunoregulatory t lymphocyte'/exp OR 'immunoregulatory t lymphocyte' OR 'regulatory t cell'/exp OR 'regulatory t cell' OR 'regulatory t lymphocytes'/exp OR 'regulatory t lymphocytes' OR 'regulatory t-lymphocytes'/exp OR 'regulatory t-lymphocytes' OR 't lymphocytes, regulatory'/exp OR 't lymphocytes, regulatory' OR 't regulatory cell'/exp OR 't regulatory cell' OR 't regulatory cells'/exp OR 't regulatory cells' OR 't regulatory lymphocyte'/exp OR 't regulatory lymphocyte' OR 't-lymphocytes, regulatory'/exp OR 't-lymphocytes, regulatory' OR 'tr1 cell'/exp OR 'tr1 cell' OR 'tr1 cells'/exp OR 'tr1 cells' OR 'treg'/exp OR 'treg' OR 'tregs'/exp OR 'tregs' OR 'regulatory t lymphocyte'/exp OR 'regulatory t lymphocyte' OR 'regulatory t-lymphocyte'/exp OR 'regulatory t-lymphocyte' OR 't-lymphocyte, regulatory' OR 'treg cell' OR 'cell, treg' OR 'cells, treg' OR 't-cells, regulatory' OR 'treg cells' OR 'cell, regulatory t' OR 'cells, regulatory t' OR 'regulatory t cells'/exp OR 'regulatory t cells' OR 't cell, regulatory' OR 't cells, regulatory' OR 'regulatory t-cell'/exp OR 'regulatory t-cell' OR 'th3 cells'/exp OR 'th3 cells' OR 'cell, th3' OR 'cells, th3' OR 'th3 cell'/exp OR 'th3 cell' OR 'suppressor t-lymphocytes, naturally-occurring' OR 'naturally-occurring suppressor t-lymphocyte' OR 'naturally-occurring suppressor t-lymphocytes' OR 'suppressor t lymphocytes, naturally occurring' OR 'suppressor t-lymphocyte, naturally-occurring' OR 'suppressor t-cells, naturally-occurring' OR 'naturally-occurring suppressor t-cell' OR 'naturally-occurring suppressor t-cells' OR 'suppressor t cells, naturally occurring' OR 'suppressor t-cell, naturally-occurring' OR 't-cell, naturally-occurring suppressor' OR 't-cells, naturally-occurring suppressor' OR 'cell, tr1' OR 'cells, tr1' OR 'cell, th17' OR 'cells, th17' OR 't helper 17 cell' OR 'th-17 cell' OR 'th 17 cell' OR 't helper 17 cells' OR 'type 17 helper t cells' OR 'th-17 cells' OR 'cell, th-17' OR 'cells, th-17' OR 'th 17 cells' OR 'type 17 helper t cell' OR 'helper cell type 17'/exp OR 'helper cell type 17' OR 't helper 17'/exp OR 't helper 17' OR 't helper type 17'/exp OR 't helper type 17' OR 'th17 cells'/exp OR 'th17 cells' OR 'th17 cell'/exp OR 'th17 cell' |
| #1 | 'uterine cervix tumor'/exp OR 'uterine cervix tumor' OR 'cervical neoplasia'/exp OR 'cervical neoplasia' OR 'cervical tumor'/exp OR 'cervical tumor' OR 'cervical tumorigenesis'/exp OR 'cervical tumorigenesis' OR 'cervical tumour'/exp OR 'cervical tumour' OR 'cervix neoplasia'/exp OR 'cervix neoplasia' OR 'cervix neoplasm'/exp OR 'cervix neoplasm' OR 'cervix neoplasms'/exp OR 'cervix neoplasms' OR 'cervix tumor'/exp OR 'cervix tumor' OR 'cervix tumorigenesis'/exp OR 'cervix tumorigenesis' OR 'cervix tumour'/exp OR 'cervix tumour' OR 'cervix uteri tumor'/exp OR 'cervix uteri tumor' OR 'neoplasia of the cervix'/exp OR 'neoplasia of the cervix' OR 'neoplasm of the cervix'/exp OR 'neoplasm of the cervix' OR 'neoplastic cervical'/exp OR 'neoplastic cervical' OR 'neoplastic cervix'/exp OR 'neoplastic cervix' OR 'tumor of the cervix'/exp OR 'tumor of the cervix' OR 'tumor of the uterine cervix'/exp OR 'tumor of the uterine cervix' OR 'tumour of the cervix'/exp OR 'tumour of the cervix' OR 'tumour of the uterine cervix'/exp OR 'tumour of the uterine cervix' OR 'uterine cervical neoplasia'/exp OR 'uterine cervical neoplasia' OR 'uterine cervical neoplasm'/exp OR 'uterine cervical neoplasm' OR 'uterine cervical neoplasms'/exp OR 'uterine cervical neoplasms' OR 'uterine cervical tumor'/exp OR 'uterine cervical tumor' OR 'uterine cervix neoplasia'/exp OR 'uterine cervix neoplasia' OR 'uterine cervix neoplasm'/exp OR 'uterine cervix neoplasm' OR 'uterine cervix tumour'/exp OR 'uterine cervix tumour' OR 'cervical neoplasm, uterine' OR 'neoplasm, uterine cervical' OR 'neoplasms, cervical' OR 'cervical neoplasm'/exp OR 'cervical neoplasm' OR 'neoplasms, cervix' OR 'neoplasm, cervix' OR 'cancer of the uterine cervix'/exp OR 'cancer of the uterine cervix' OR 'cancer of the cervix'/exp OR 'cancer of the cervix' OR 'cervical cancer'/exp OR 'cervical cancer' OR 'cancer, cervical' OR 'cervical cancers' OR 'uterine cervical cancer'/exp OR 'uterine cervical cancer' OR 'cancer, uterine cervical' OR 'cervical cancer, uterine' OR 'uterine cervical cancers' OR 'cancer of cervix' OR 'cervix cancer'/exp OR 'cervix cancer' OR 'cancer, cervix' OR 'uterine cervical dysplasia'/exp OR 'uterine cervical dysplasia' OR 'cervical dysplasia, uterine' OR 'dysplasia of cervix uteri'/exp OR 'dysplasia of cervix uteri' OR 'cervix uteri dysplasia' OR 'cervix uteri dysplasias' OR 'cervical intraepithelial neoplasia'/exp OR 'cervical intraepithelial neoplasia' OR 'cervical intraepithelial neoplasms' OR 'cervical intraepithelial neoplasm'/exp OR 'cervical intraepithelial neoplasm' OR 'intraepithelial neoplasm, cervical' OR 'intraepithelial neoplasms, cervical' OR 'neoplasm, cervical intraepithelial' OR 'neoplasms, cervical intraepithelial' OR 'intraepithelial neoplasia, cervical' OR 'neoplasia, cervical intraepithelial' OR 'cervical dysplasia'/exp OR 'cervical dysplasia' OR 'cervical dysplasias' OR 'dysplasia, cervical' OR 'cervix dysplasia'/exp OR 'cervix dysplasia' OR 'dysplasia, cervix'/exp OR 'dysplasia, cervix' OR 'cervical intraepithelial neoplasia, grade iii'/exp OR 'cervical intraepithelial neoplasia, grade iii' OR 'cervix dysplasia, uterine'/exp OR 'cervix dysplasia, uterine' OR 'dysplasia of the uterine cervix'/exp OR 'dysplasia of the uterine cervix' OR 'uterine cervix squamous dysplasia'/exp OR 'uterine cervix squamous dysplasia' OR 'uterine cervix dysplasia'/exp OR 'uterine cervix dysplasia' |

**The search terms in WEB OF SCIENCE**

| 1 | (((((((((((((((((((((((((((((((((((((((((((((((((((((((((((((((((((((TS=(uterine cervix tumor)) OR TS=(cervical neoplasia)) OR TS=(cervical neoplasm)) OR TS=(cervical tumor)) OR TS=(cervical tumorigenesis)) OR TS=(cervical tumour)) OR TS=(cervix neoplasia)) OR TS=(cervix neoplasm)) OR TS=(cervix neoplasms)) OR TS=(cervix tumor)) OR TS=(cervix tumorigenesis)) OR TS=(cervix tumour)) OR TS=(cervix uteri tumor)) OR TS=(neoplasia of the cervix)) OR TS=(neoplasm of the cervix)) OR TS=(neoplastic cervical)) OR TS=(neoplastic cervix)) OR TS=(tumor of the cervix)) OR TS=(tumor of the uterine cervix)) OR TS=(tumour of the cervix)) OR TS=(tumour of the uterine cervix)) OR TS=(uterine cervical neoplasia)) OR TS=(uterine cervical neoplasm)) OR TS=(uterine cervical neoplasms)) OR TS=(uterine cervical tumor)) OR TS=(uterine cervix neoplasia)) OR TS=(uterine cervix neoplasm)) OR TS=(uterine cervix tumour)) OR TS=(Cervical Neoplasm, Uterine)) OR TS=(Neoplasm, Uterine Cervical)) OR TS=(Neoplasms, Cervical)) OR TS=(Cervical Neoplasm)) OR TS=(Neoplasms, Cervix)) OR TS=(Neoplasm, Cervix)) OR TS=(Cancer of the Uterine Cervix)) OR TS=(Cancer of the Cervix)) OR TS=(Cervical Cancer)) OR TS=(Cancer, Cervical)) OR TS=(Cervical Cancers)) OR TS=(Uterine Cervical Cancer)) OR TS=(Cancer, Uterine Cervical)) OR TS=(Cervical Cancer, Uterine)) OR TS=(Uterine Cervical Cancers)) OR TS=(Cancer of Cervix)) OR TS=(Cervix Cancer)) OR TS=(Cancer, Cervix)) OR TS=(Uterine Cervical Dysplasia)) OR TS=(Cervical Dysplasia, Uterine)) OR TS=(Dysplasia of Cervix Uteri)) OR TS=(Cervix Uteri Dysplasia)) OR TS=(Cervix Uteri Dysplasias)) OR TS=(Cervical Intraepithelial Neoplasia)) OR TS=(Cervical Intraepithelial Neoplasms)) OR TS=(Cervical Intraepithelial Neoplasm)) OR TS=(Intraepithelial Neoplasm, Cervical)) OR TS=(Intraepithelial Neoplasms, Cervical)) OR TS=(Neoplasm, Cervical Intraepithelial)) OR TS=(Neoplasms, Cervical Intraepithelial)) OR TS=(Intraepithelial Neoplasia, Cervical)) OR TS=(Neoplasia, Cervical Intraepithelial)) OR TS=(Cervical Dysplasia)) OR TS=(Cervical Dysplasias)) OR TS=(Dysplasia, Cervical)) OR TS=(Cervix Dysplasia)) OR TS=(Dysplasia, Cervix)) OR TS=(Cervical Intraepithelial Neoplasia, Grade III)) OR TS=(cervix dysplasia, uterine)) OR TS=(dysplasia of the uterine cervix)) OR TS=(uterine cervix squamous dysplasia)) OR TS=(uterine cervix dysplasia) |
| --- | --- |
| 2 | ((((((((((((((((((((((((((((((((((((((((((((((((((((((((((((((((TS=(immunoregulatory T cell)) OR TS=(immunoregulatory T cells)) OR TS=(immunoregulatory T lymphocyte)) OR TS=(regulatory T cell)) OR TS=(regulatory T cells)) OR TS=(regulatory T lymphocytes)) OR TS=(regulatory t-lymphocytes)) OR TS=(T lymphocytes, regulatory)) OR TS=(T regulatory cell)) OR TS=(T regulatory cells)) OR TS=(T regulatory lymphocyte)) OR TS=(T-lymphocytes, regulatory)) OR TS=(Tr1 cell)) OR TS=(Tr1 cells)) OR TS=(Treg)) OR TS=(Tregs)) OR TS=(regulatory T lymphocyte)) OR TS=(Regulatory T-Lymphocyte)) OR TS=(T-Lymphocyte, Regulatory)) OR TS=(Treg Cell)) OR TS=(Cell, Treg)) OR TS=(Cells, Treg)) OR TS=(T-Cells, Regulatory)) OR TS=(Treg Cells)) OR TS=(Cell, Regulatory T)) OR TS=(Cells, Regulatory T)) OR TS=(Regulatory T Cells)) OR TS=(T Cell, Regulatory)) OR TS=(T Cells, Regulatory)) OR TS=(Regulatory T-Cell)) OR TS=(Th3 Cells)) OR TS=(Cell, Th3)) OR TS=(Cells, Th3)) OR TS=(Th3 Cell)) OR TS=(Suppressor T-Lymphocytes, Naturally-Occurring)) OR TS=(Naturally-Occurring Suppressor T-Lymphocyte)) OR TS=(Naturally-Occurring Suppressor T-Lymphocytes)) OR TS=(Suppressor T Lymphocytes, Naturally Occurring)) OR TS=(Suppressor T-Lymphocyte, Naturally-Occurring)) OR TS=(Suppressor T-Cells, Naturally-Occurring)) OR TS=(Naturally-Occurring Suppressor T-Cell)) OR TS=(Naturally-Occurring Suppressor T-Cells)) OR TS=(Suppressor T Cells, Naturally Occurring)) OR TS=(T-Cell, Naturally-Occurring Suppressor)) OR TS=(T-Cells, Naturally-Occurring Suppressor)) OR TS=(Cell, Tr1)) OR TS=(Cells, Tr1)) OR TS=(Th17 Cell)) OR TS=(Cell, Th17)) OR TS=(Cells, Th17)) OR TS=(T Helper 17 Cell)) OR TS=(TH-17 Cell)) OR TS=(TH 17 Cell)) OR TS=(T Helper 17 Cells)) OR TS=(Type 17 Helper T Cells)) OR TS=(TH-17 Cells)) OR TS=(Cell, TH-17)) OR TS=(Cells, TH-17)) OR TS=(TH 17 Cells)) OR TS=(Type 17 Helper T Cell)) OR TS=(helper cell type 17)) OR TS=(T helper 17)) OR TS=(T helper type 17)) OR TS=(Th17 cells)) OR TS=(Th17 cell) |
| 3 | #1 AND #2 |

# Supplementary Table 1

# PRISMA 2020 Main Checklist

| **Topic** | **No.** | **Item** | **Location where item is reported** |
| --- | --- | --- | --- |
| **TITLE** |  |  |  |
| **Title** | 1 | Identify the report as a systematic review. | Title |
| **ABSTRACT** |  |  |  |
| **Abstract** | 2 | See the PRISMA 2020 for Abstracts checklist |  |
| **INTRODUCTION** |  |  |  |
| **Rationale** | 3 | Describe the rationale for the review in the context of existing knowledge. | Paragraph 1-4 of Introduction |
| **Objectives** | 4 | Provide an explicit statement of the objective(s) or question(s) the review addresses. | Paragraph 5 of  Introduction |
| **METHODS** |  |  |  |
| **Eligibility criteria** | 5 | Specify the inclusion and exclusion criteria for the review and how studies were grouped for the syntheses. | Study selection and quality assessment |
| **Information sources** | 6 | Specify all databases, registers, websites, organisations, reference lists and other sources searched or consulted to identify studies. Specify the date when each source was last searched or consulted. | Search strategy and information sources |
| **Search strategy** | 7 | Present the full search strategies for all databases, registers and websites, including any filters and limits used. | Search strategy and information sources |
| **Selection process** | 8 | Specify the methods used to decide whether a study met the inclusion criteria of the review, including how many reviewers screened each record and each report retrieved, whether they worked independently, and if applicable, details of automation tools used in the process. | Search strategy and information sources |
| **Data collection process** | 9 | Specify the methods used to collect data from reports, including how many reviewers collected data from each report, whether they worked independently, any processes for obtaining or confirming data from study investigators, and if applicable, details of automation tools used in the process. | Data extraction |
| **Data items** | 10a | List and define all outcomes for which data were sought. Specify whether all results that were compatible with each outcome domain in each study were sought (e.g. for all measures, time points, analyses), and if not, the methods used to decide which results to collect. | Data extraction |
|  | 10b | List and define all other variables for which data were sought (e.g. participant and intervention characteristics, funding sources). Describe any assumptions made about any missing or unclear information. | Data extraction |
| **Study risk of bias assessment** | 11 | Specify the methods used to assess risk of bias in the included studies, including details of the tool(s) used, how many reviewers assessed each study and whether they worked independently, and if applicable, details of automation tools used in the process. | Assessment of risk of bias and sensitivity analysis |
| **Effect measures** | 12 | Specify for each outcome the effect measure(s) (e.g. risk ratio, mean difference) used in the synthesis or presentation of results. | Data synthesis |
| **Synthesis methods** | 13a | Describe the processes used to decide which studies were eligible for each synthesis (e.g. tabulating the study intervention characteristics and comparing against the planned groups for each synthesis (item 5)). | Data extraction |
|  | 13b | Describe any methods required to prepare the data for presentation or synthesis, such as handling of missing summary statistics, or data conversions. | Data extraction |
|  | 13c | Describe any methods used to tabulate or visually display results of individual studies and syntheses. | Data synthesis |
|  | 13d | Describe any methods used to synthesize results and provide a rationale for the choice(s). If meta-analysis was performed, describe the model(s), method(s) to identify the presence and extent of statistical heterogeneity, and software package(s) used. | Paragraph 1 of Data synthesis section |
|  | 13e | Describe any methods used to explore possible causes of heterogeneity among study results (e.g. subgroup analysis, meta-regression). | Paragraph 1 of Data synthesis section |
|  | 13f | Describe any sensitivity analyses conducted to assess robustness of the synthesized results. | Assessment of risk of bias and sensitivity analysis |
| **Reporting bias assessment** | 14 | Describe any methods used to assess risk of bias due to missing results in a synthesis (arising from reporting biases). | Assessment of risk of bias and sensitivity analysis |
| **Certainty assessment** | 15 | Describe any methods used to assess certainty (or confidence) in the body of evidence for an outcome. | Paragraph 2 of Study selection and quality assessment section |
| **RESULTS** |  |  |  |
| **Study selection** | 16a | Describe the results of the search and selection process, from the number of records identified in the search to the number of studies included in the review, ideally using a flow diagram. | Paragraph 1 of Study characteristics section and Figure 1 |
|  | 16b | Cite studies that might appear to meet the inclusion criteria, but which were excluded, and explain why they were excluded. | N/A |
| **Study characteristics** | 17 | Cite each included study and present its characteristics. | Study characteristics and Table 1 |
| **Risk of bias in studies** | 18 | Present assessments of risk of bias for each included study. | Paragraph 1 of Publication bias and sensitivity analysis section |
| **Results of individual studies** | 19 | For all outcomes, present, for each study: (a) summary statistics for each group (where appropriate) and (b) an effect estimate and its precision (e.g. confidence/credible interval), ideally using structured tables or plots. | Figure 2, 3 and 5 |
| **Results of syntheses** | 20a | For each synthesis, briefly summarise the characteristics and risk of bias among contributing studies. | Study characteristics |
|  | 20b | Present results of all statistical syntheses conducted. If meta-analysis was done, present for each the summary estimate and its precision (e.g. confidence/credible interval) and measures of statistical heterogeneity. If comparing groups, describe the direction of the effect. | “The prognostic value of Treg and Th17 cells” and” The value of Treg and Th17 cells, and Th17/Treg ratio in clinical features of CC patients” sections |
|  | 20c | Present results of all investigations of possible causes of heterogeneity among study results. | Subgroup analysis |
|  | 20d | Present results of all sensitivity analyses conducted to assess the robustness of the synthesized results. | Paragraph2 of Publication bias and sensitivity analysis section |
| **Reporting biases** | 21 | Present assessments of risk of bias due to missing results (arising from reporting biases) for each synthesis assessed. | Line 152-156 |
| **Certainty of evidence** | 22 | Present assessments of certainty (or confidence) in the body of evidence for each outcome assessed. | Paragraph 2 of Study characteristics section and 3 Supplementary Table 2 |
| **DISCUSSION** |  |  |  |
| **Discussion** | 23a | Provide a general interpretation of the results in the context of other evidence. | Paragraph 1-3 of Discussion |
|  | 23b | Discuss any limitations of the evidence included in the review. | Paragraph 4 of Discussion |
|  | 23c | Discuss any limitations of the review processes used. | Paragraph 4 of Discussion |
|  | 23d | Discuss implications of the results for practice, policy, and future research. | Paragraph 5-6 of Discussion |
| **OTHER INFORMATION** |  |  |  |
| **Registration and protocol** | 24a | Provide registration information for the review, including register name and registration number, or state that the review was not registered. | Paragraph 1 of Materials and methods |
|  | 24b | Indicate where the review protocol can be accessed, or state that a protocol was not prepared. | Paragraph 1 of Materials and methods |
|  | 24c | Describe and explain any amendments to information provided at registration or in the protocol. | N/A |
| **Support** | 25 | Describe sources of financial or non-financial support for the review, and the role of the funders or sponsors in the review. | Funding Information |
| **Competing interests** | 26 | Declare any competing interests of review authors. | Conflict of Interest |
| **Availability of data, code and other materials** | 27 | Report which of the following are publicly available and where they can be found: template data collection forms; data extracted from included studies; data used for all analyses; analytic code; any other materials used in the review. | Data availability statement |

#####

# PRIMSA Abstract Checklist

| **Topic** | **No.** | **Item** | **Reported?** |
| --- | --- | --- | --- |
| **TITLE** |  |  |  |
| **Title** | 1 | Identify the report as a systematic review. | Yes |
| **BACKGROUND** |  |  |  |
| **Objectives** | 2 | Provide an explicit statement of the main objective(s) or question(s) the review addresses. | Yes |
| **METHODS** |  |  |  |
| **Eligibility criteria** | 3 | Specify the inclusion and exclusion criteria for the review. | Yes |
| **Information sources** | 4 | Specify the information sources (e.g. databases, registers) used to identify studies and the date when each was last searched. | Yes |
| **Risk of bias** | 5 | Specify the methods used to assess risk of bias in the included studies. | Yes |
| **Synthesis of results** | 6 | Specify the methods used to present and synthesize results. | Yes |
| **RESULTS** |  |  |  |
| **Included studies** | 7 | Give the total number of included studies and participants and summarise relevant characteristics of studies. | Yes |
| **Synthesis of results** | 8 | Present results for main outcomes, preferably indicating the number of included studies and participants for each. If meta-analysis was done, report the summary estimate and confidence/credible interval. If comparing groups, indicate the direction of the effect (i.e. which group is favoured). | Yes |
| **DISCUSSION** |  |  |  |
| **Limitations of evidence** | 9 | Provide a brief summary of the limitations of the evidence included in the review (e.g. study risk of bias, inconsistency and imprecision). | Yes |
| **Interpretation** | 10 | Provide a general interpretation of the results and important implications. | Yes |
| **OTHER** |  |  |  |
| **Funding** | 11 | Specify the primary source of funding for the review. | No |
| **Registration** | 12 | Provide the register name and registration number. | Yes |

*From:* Page MJ, McKenzie JE, Bossuyt PM, Boutron I, Hoffmann TC, Mulrow CD, et al. The PRISMA 2020 statement: an updated guideline for reporting systematic reviews. MetaArXiv. 2020, September 14. DOI: 10.31222/osf.io/v7gm2. For more information, visit: [www.prisma-statement.org](file:///D:\Downloads\www.prisma-statement.org)

# Supplementary Table 2

**Table A** NOS quality assessment of cohort studies

| **study ID** | **Selection** | | | | **Comparability** | **Outcome** | | | **scores** |
| --- | --- | --- | --- | --- | --- | --- | --- | --- | --- |
|  | **1** | **2** | **3** | **4** | **5** | **6** | **7** | **8** |  |
| Jordanova 2008 | ★ | ★ | ★ | ★ | ★ | ★ | ★ |  | 7 |
| Shah 2010 | ★ | ★ | ★ | ★ |  | ★ | ★ | ★ | 7 |
| Gorter 2015 | ★ | ★ | ★ | ★ |  | ★ | ★ |  | 6 |
| Yang 2019 | ★ | ★ | ★ | ★ | ★ | ★ | ★ | ★ | 8 |
| Lu 2020 | ★ | ★ | ★ | ★ |  | ★ | ★ |  | 6 |
| Zhang 2022 | ★ | ★ | ★ | ★ |  | ★ | ★ | ★ | 7 |
| Ni 2022 | ★ | ★ | ★ | ★ | ★ | ★ | ★ |  | 7 |
| Yang 2023 | ★ | ★ | ★ | ★ |  | ★ | ★ |  | 6 |
| Li 2021 | ★ | ★ | ★ | ★ |  | ★ | ★ |  | 6 |

**Table B** AHRQ quality assessment of cross-sectional studies

| **study** | **1** | **2** | **3** | **4** | **5** | **6** | **7** | **8** | **9** | **10** | **11** | **score** |
| --- | --- | --- | --- | --- | --- | --- | --- | --- | --- | --- | --- | --- |
| Zhang 2011 | Y | Y | N | U | Y | Y | Y | N | U | Y | U | 6 |
| Zhang 2015 | Y | Y | N | U | Y | Y | Y | N | U | Y | U | 6 |
| Lin 2020 | Y | Y | N | U | Y | Y | Y | U | U | Y | U | 6 |
| Xue 2018 | Y | Y | Y | U | Y | Y | Y | U | U | Y | U | 7 |
| Lin 2019 | Y | Y | N | U | Y | Y | Y | U | U | Y | U | 6 |
| Yu 2015 | Y | Y | Y | U | Y | Y | Y | U | U | Y | U | 7 |
| Hou 2012 | Y | Y | N | U | Y | Y | Y | U | U | Y | U | 6 |
| Wang 2023 | Y | Y | Y | U | Y | Y | Y | Y | U | Y | U | 8 |
| Chen 2013 | Y | Y | Y | U | Y | Y | Y | U | U | Y | U | 7 |
| Ohno 2020 | Y | Y | Y | U | Y | Y | Y | U | U | Y | U | 7 |
| Chen 2019 | Y | Y | Y | U | Y | Y | Y | U | U | Y | U | 7 |
| Adurthi 2008 | Y | Y | N | U | Y | Y | Y | N | U | Y | U | 6 |

# Supplementary Figure 1


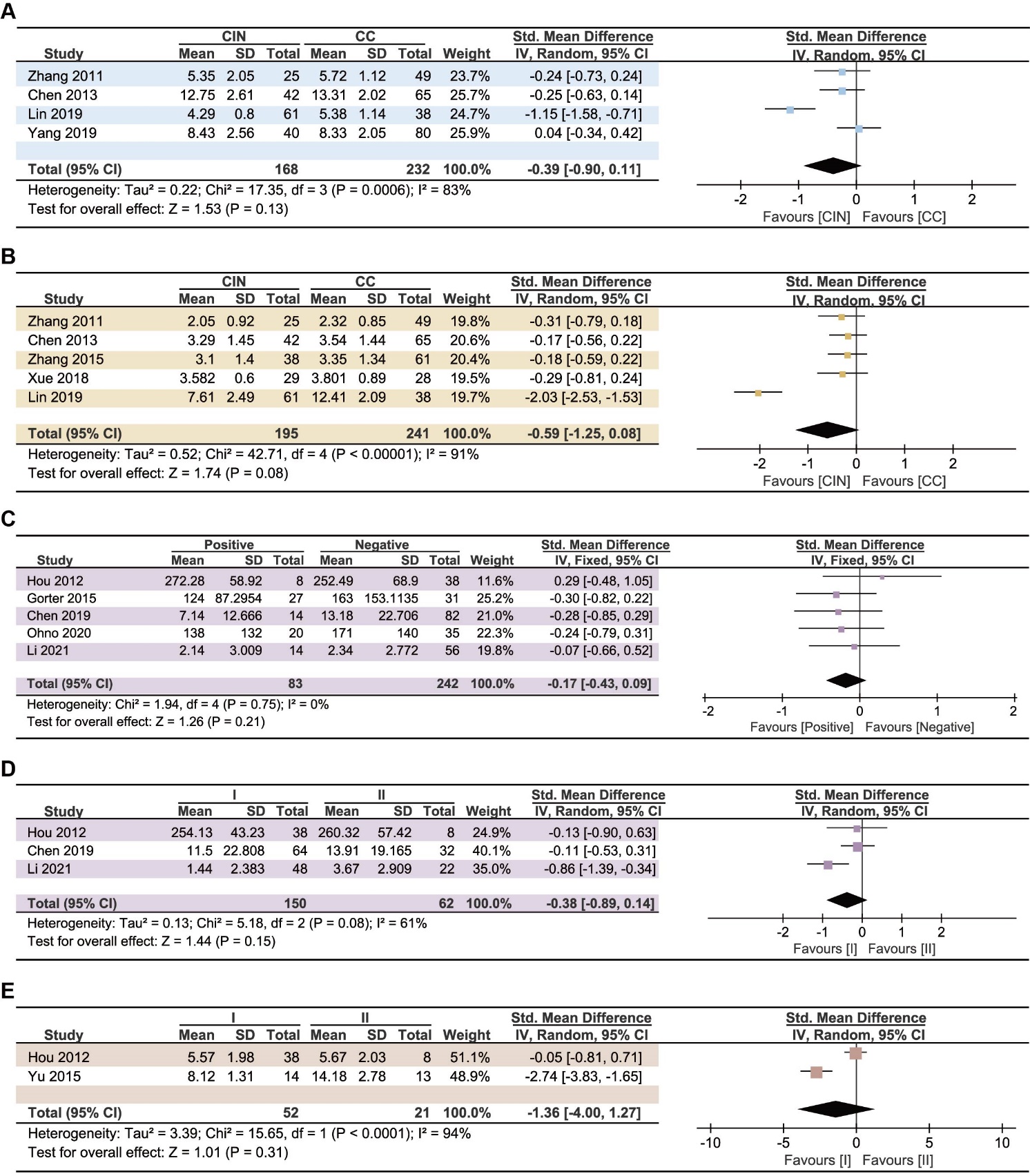


**Supplementary Figure 1.** Forest plots assessing the association between Treg, Th17 and CC patients’ clinical features. No statistical association was found in these analyses. **A** Differences of Treg cells in PB between CIN and CC patients. **B** Differences of Th17 cells in PB between CIN and CC patients. **C** Relationship between Treg cells in tumor tissue and lymphoid metastases in CC patients. **D** Relationship between Treg cells in tumor tissue and clinical stage in CC patients. **E** Relationship between Th17 cells in tumor tissue and clinical stage in CC patients.
